# Complete Mitochondrial Genome Sequence of the Phytopathogenic Basidiomycete Ganoderma boninense Strain G3

**DOI:** 10.1128/MRA.00968-18

**Published:** 2019-01-31

**Authors:** Condro Utomo, Zulfikar Achmad Tanjung, Redi Aditama, Rika Fithri Nurani Buana, Antonius Dony Madu Pratomo, Reno Tryono, Tony Liwang

**Affiliations:** a Department of Biotechnology, Plant Production and Biotechnology Division, PT SMART Tbk, Bogor, Indonesia; b Section of Bioinformatics, PT SMART Tbk, Bogor, Indonesia; c Section of Microbiome Technology, PT SMART Tbk, Bogor, Indonesia; d Section of Clonal Technology, PT SMART Tbk, Bogor, Indonesia; e Section of Genetic Engineering, PT SMART Tbk, Bogor, Indonesia; Vanderbilt University

## Abstract

Here, we report the complete sequence of the mitochondrial (mt) genome of Ganoderma boninense that assembled as a single circular double-stranded DNA (dsDNA) of 86,549 bp with a G+C content of 26.81%. Genome annotation identified genes that encode 15 conserved proteins, 27 tRNAs, small and large rRNA subunits, 4 hypothetical proteins, and 5 homing endonucleases.

## ANNOUNCEMENT

The oil palm (Elaeis guineensis Jacq) is the world’s most efficient oil-producing crop. Indonesia and Malaysia are major producers, but both are facing an economically important disease called basal stem rot disease that is caused by a basidiomycete fungus, Ganoderma boninense (1). A draft genome sequence of this phytopathogen was recently published, enabling further investigation of its mitochondrial (mt) genome (2).

To obtain the mt genome, all 495 genomic DNA scaffolds of G. boninense under the NCBI GenBank accession number PJEW00000000 were aligned to the mt genome of its closely related species, G. lucidum, derived from a sequence under the EMBL GenBank accession number HF570115, which was used as the reference (3). The alignment was done using the NCBI BLAST 2.6.0+ program with the following parameter settings: gapopen (5), gapextend (2), reward (1), and penalty (-2). The result showed that scaffold number 204 was the best hit with the highest alignment score of 8,468 hits and 97% nucleotide sequence similarity with the mt genome of G. lucidum. Scaffold number 204 has size of 139,127 bp; however, the first 52,578 bp at the 5′ end of the contig are identical to the last 52,578 bp of the 3′ end of the contig, suggesting that this contig corresponded to a circular genome. Subsequently, a nongap circular contig of 86,549 bp with a G+C content of 26.81% was built using Geneious R.10 software (4). Gene annotation was done with MFannot using the NCBI translation (5–7).

The G. boninense mt genome contains 15 conserved proteins, 27 tRNAs, small and large rRNA subunits, 4 hypothetical proteins, and 5 homing endonucleases. The genes comprise cytochrome *c* oxidase subunits 1 to 3 (*cox1*, *cox2*, and *cox3*), apocytochrome *b* (*cob*), NADH dehydrogenase subunits 1 to 6 (*nad1*, *nad2*, *nad3*, *nad4*, *nad4L*, *nad5*, and *nad6*), ATP synthase subunits 6 to 9 (*atp6*, *atp8*, and *atp9*), and ribosomal protein (*rps3*). All tRNA- and rRNA-encoding genes (27 tRNAs, small and large rRNA subunits) and 4 hypothetical proteins were found on the positive strand and were clockwise oriented, except for trnW-CCA, which was found in the negative strand and was counterclockwise oriented. In addition, 5 homing endonucleases with LAGLIDADG motif patterns were identified.

### Data availability.

This mt genome shotgun project has been deposited at the NCBI database under the BioProject accession number PRJNA421251. In addition, all raw reads to build scaffold 204 were also deposited under the SRA run number SRR8351970.
